# Pulsatile cerebral paraarterial flow by peristalsis, pressure and directional resistance

**DOI:** 10.1186/s12987-023-00445-0

**Published:** 2023-06-08

**Authors:** M. Keith Sharp

**Affiliations:** Department of Mechanical Engineering, University of Louisville, 200 Sackett Hall, Louisville, KY 40292 USA

**Keywords:** Brain, Glymphatic flow, Peristaltic flow, Perivascular, Paravascular

## Abstract

**Background:**

A glymphatic system has been proposed that comprises flow that enters along cerebral paraarterial channels between the artery wall and the surrounding glial layer, continues through the parenchyma, and then exits along similar paravenous channels. The mechanism driving flow through this system is unclear. The pulsatile (oscillatory plus mean) flow measured in the space surrounding the middle cerebral artery (MCA) suggests that peristalsis created by intravascular blood pressure pulses is a candidate for the paraarterial flow in the subarachnoid spaces. However, peristalsis is ineffective in driving significant mean flow when the amplitude of channel wall motion is small, as has been observed in the MCA artery wall. In this paper, peristalsis in combination with two additional mechanisms, a longitudinal pressure gradient and directional flow resistance, is evaluated to match the measured MCA paraarterial oscillatory and mean flows.

**Methods:**

Two analytical models are used that simplify the paraarterial branched network to a long continuous channel with a traveling wave in order to maximize the potential effect of peristalsis on the mean flow. The two models have parallel-plate and annulus geometries, respectively, with and without an added longitudinal pressure gradient. The effect of directional flow resistors was also evaluated for the parallel-plate geometry.

**Results:**

For these models, the measured amplitude of arterial wall motion is too large to cause the small measured amplitude of oscillatory velocity, indicating that the outer wall must also move. At a combined motion matching the measured oscillatory velocity, peristalsis is incapable of driving enough mean flow. Directional flow resistance elements augment the mean flow, but not enough to provide a match. With a steady longitudinal pressure gradient, both oscillatory and mean flows can be matched to the measurements.

**Conclusions:**

These results suggest that peristalsis drives the oscillatory flow in the subarachnoid paraarterial space, but is incapable of driving the mean flow. The effect of directional flow resistors is insufficient to produce a match, but a small longitudinal pressure gradient is capable of creating the mean flow. Additional experiments are needed to confirm whether the outer wall also moves, as well as to validate the pressure gradient.

## Background

**Fig. 1:**
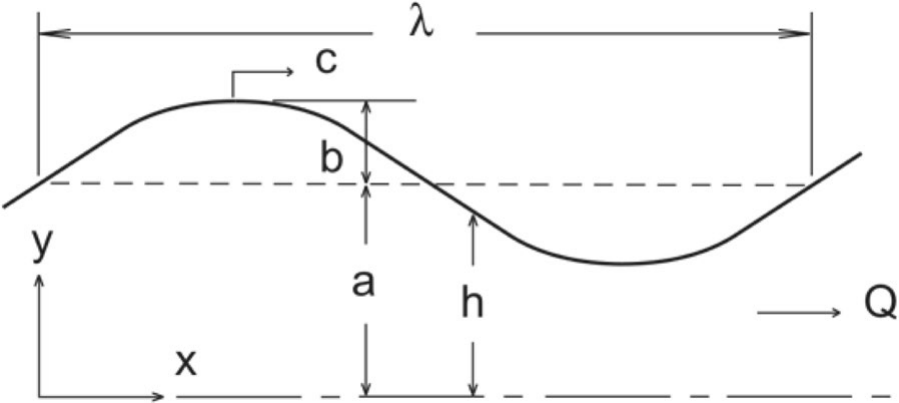
Geometric parameters of the peristalitc wall motion

A glymphatic circulation in the brain has been proposed that involves transport through cerebral paraarterial channels, the parenchyma and paravenous channels ([1], for reviews, see [2, 3]). To confirm this hypothesis, it is essential that mechanisms driving transport through each section be identified and validated [4, 5]. Transport of solutes need not involve net bulk flow of fluid. Alternatively, shear-augmented dispersion in the absence of net flow might contribute to or entirely explain the transport [6]. For the paraarterial channels, an obvious candidate driver of flow in the subarachnoid paraarterial space is peristalsis associated with displacement of the arterial wall during the blood pressure pulse. Recent in vivo measurements of arterial displacement and flow in paravascular channels surrounding the middle cerebral artery (MCA) [7] provide validation data for modelling efforts to test the plausibility of peristaltic flow. Mestre, et al. [7] found a mean flow velocity of 18.7 μm/s in a channel with gap of *a* = 44 μm surrounding an artery of radius 22 μm. Mestre, et al. did not report oscillatory velocity, but Ladrón-de-Guevara, et al. [8] give the ratio of temporal peak, spatial root-mean-square oscillatory velocity to mean velocity as 0.53. For a parabolic profile, the ratio of mean to root-mean-square is 5/4, thus the amplitude of the spatial mean of oscillatory velocity is estimated as 18.7 * 5/4 = 12.3 μm/s. From Mestre, et al. [7] Fig. 3e, artery diameter amplitude normalized by the baseline diameter is estimated as 0.008, which gives a radial amplitude of *b* = 0.176 μm and amplitude ratio $\phi \equiv \frac{b}{a}$= 0.004 (see Fig. 1).

The MCA channels represent the initial conduits of the proposed glymphatic circulation, that includes a branched network of paraarterial channels, interstitial flow through the parenchyma and outflow through a branched network of paravenous channels. Faghih and Sharp [9] modelled flow in the paraarterial network and Vinje, et al*.* [10] analysed the more comprehensive system, but both assumed steady flow only.

Pulsatile flow models have been simplified in two categories, either a single channel (without branching) longer than the pulsatile wavelength representing the paraarterial network, or a section of a proximal paraarterial channel shorter than the wavelength. When the channel length is much shorter than the traveling wave, the flow approaches that of a squeeze flow caused by synchronous wall motion, which produces little to no mean flow. Accordingly, a number of investigators using this type of simplified model found insignificant mean flow (*e.g*., [11–13]).

A model longer than the wavelength was used by Wang and Olbricht [14] to calculate mean flow by peristalsis and a pressure gradient caused by injection, as a function of wall displacement and channel permeability. Romano, et al. [15] added permeable, Hookean brain tissue to model flow through the glial boundary. Ranges of geometric parameters and elasticity and permeability were tested, but only one constant longitudinal pressure difference was used. They found that a phase shift was necessary between glial and arterial wall displacements for steady streaming to occur. Depending on elasticity and permeability, flow can either enter or exit the brain. The velocities are small (− 2.25–0.4 μm/s for their ranges of parameters) compared to that measured by Mestre, et al. [7], and may characterize the error associated with neglecting phase differences in favour of simpler models.

Reducing the branched network of paraarterial channels to a single channel of the same length and constant mean cross-sectional geometry involves assumptions that may significantly influence the resulting flow. For instance, such a simplification neglects resistance associated with flow through branches. It also ignores wave reflection at branches, *i.e.*, it assumes that the traveling wave is entirely transmitted along the channel without reflection. Reductions in wave amplitude with distance along the network, associated with decreasing pulse pressure, are also ignored. Decreased permeability of the channel associated with possible increase in structure within distal branches is likewise neglected. Nonetheless, the simplified model is attractive for its relative ease of solution and, in this case, for the upper limit of mean flow that it likely represents. This study uses this “long” model to evaluate whether peristalsis alone is capable of matching the Mestre, et al. [7] oscillatory and mean flow, as well as whether two additional mechanisms—a steady longitudinal pressure gradient and directional flow resistors—could be added to reproduce these in vivo flows.

In this paper, several mathematical solutions are applied, including peristalsis with and without a longitudinal pressure gradient in parallel-plate [16] and annulus [14] geometries, and peristalsis with directional flow resistors in the parallel-plate geometry. The latter solution is that of Sharp, et al. [17] with the resistors flexing in the direction of blood flow to promote forward flow. These solutions are summarized in the Methods sections. More details can be found in Sharp, et al. [17] or in the original papers.

## Methods

### Parallel-plate geometry

Jaffrin & Shapiro [16] developed a solution for nonporous flow in a cartesian 2D gap. With a thin gap *a* compared to vessel radius *A*, *a*/*A* <  < 1, the flow is modeled as that between symmetrically oscillating parallel plates (Fig. 1). The measured gap to artery radius ratio of 2.0 [7] suggests that the cartesian geometry is a compromise. Nonetheless, because this geometry overestimates the mean flow that can be created by peristalsis, it is conservative for the purposes of this study.

The flow can be described by the Navier–Stokes equation. However, with gap height of *a* = 44 μm [7] and estimated wavelength of λ = 0.1 m [18], the wavenumber $\mathrm{\alpha }\equiv \frac{2\pi a}{\lambda }=$ 2.77E-9 is small, thus the convective terms are negligible. With estimated wavespeed of *c* = 1 m/s [14] and kinematic viscosity of ν= 6.97E-7 m^2^/s [7], the characteristic Reynolds number $R\equiv \frac{ac\alpha }{\nu }=$ 1.75E-13 is small, thus the inertial terms are small. These small values allow a simplification to Stokes flow.

Boundary conditions are ${u}_{y}=0$ at the centerline $y=0$ (by flow symmetry), and $u=0$ at the wall $y=h$ (no slip). The solution involves transformation to the wave frame in which, by continuity, flow rate must be constant. Finally, the flow rate in the laboratory frame becomes

1 $$\overline{Q }=\frac{3{\phi }^{2}}{2+{\phi }^{2}}-\frac{1}{3\pi }\frac{{\left(1-{\phi }^{2}\right)}^{5/2}}{2+{\phi }^{2}}\Delta {P}_{\lambda }$$

Equation 1 establishes the pressure drop versus flow relationship for the peristaltic pump. The first term is a function only of the amplitude ratio ϕ and represents the forward pumping due to peristalsis. The second term, which goes to zero as the amplitude ratio approaches one (complete occlusion of the channel at the valleys in the peristaltic wave), represents the back leakage due to an unfavorable (positive) pressure gradient or augmented flow due to a favorable (negative) pressure gradient.

The mean velocity across the cross section is

2 $$\overline{U }\left(\tau \right)\equiv \frac{\overline{u }\left(\tau \right)}{c}=\frac{1}{H\left(\tau \right)}\left(\overline{Q }+H\left(\tau \right)-1\right)$$

### Annular geometry

Wang & Olbricht [14] used a Darcy model for flow in porous media in an annular space with an oscillating inner wall at radius $h\left(t\right)$. The instantaneous flow rate is

3 $$q\left(t\right)=-\frac{2\pi\Phi {cR}_{2}^{2}\left(\frac{\kappa \Delta {P}_{\lambda }}{\Phi c\mu \lambda }+1\right)}{\frac{1}{\sqrt{{\left(1-\frac{{R}_{1}}{{R}_{2}}\right)}^{2}-{\left(\frac{b}{{R}_{2}}\right)}^{2}}}+\frac{1}{\sqrt{{\left(1+\frac{{R}_{1}}{{R}_{2}}\right)}^{2}-{\left(\frac{b}{{R}_{2}}\right)}^{2}}}}+\pi\Phi c\left({R}_{2}^{2}-{h\left(t\right)}^{2}\right)$$

where Φ is the porosity, *R*_*1*_ and *R*_*2*_ are the mean inner and outer radii, *b* is the amplitude of inner wall displacement, and κ is the permeability. This flow rate is largest when $h={R}_{1}-b$ with *h* ≥ 0 (the wall cannot displace farther than the center of the artery)

For this model, the nondimensional mean velocity becomes

4 $$\overline{U }\equiv \overline{Q }=\frac{\Phi {R}_{2}^{2}}{{R}_{2}^{2}-{R}_{1}^{2}}\left[-\frac{2\left(\frac{\kappa \Delta {P}_{\lambda }}{\Phi c\mu \lambda }+1\right)}{\frac{1}{\sqrt{{\left(1-\frac{{R}_{1}}{{R}_{2}}\right)}^{2}-{\left(\frac{b}{{R}_{2}}\right)}^{2}}}+\frac{1}{\sqrt{{\left(1+\frac{{R}_{1}}{{R}_{2}}\right)}^{2}-{\left(\frac{b}{{R}_{2}}\right)}^{2}}}}+1-{\left(\frac{{R}_{1}}{{R}_{2}}\right)}^{2}-\frac{1}{2}{\left(\frac{b}{{R}_{2}}\right)}^{2}\right]$$

To represent a nonporous channel, Φ  = 1 and the Darcy permeability is set to that for Poiseuille flow

5 $$\kappa =\frac{{R}_{2}^{2}}{8}\left[1+{\left(\frac{{R}_{1}}{{R}_{2}}\right)}^{2}-\frac{1-{\left(\frac{{R}_{1}}{{R}_{2}}\right)}^{2}}{ln\left(\frac{{R}_{2}}{{R}_{1}}\right)}\right]$$

### Parallel-plate geometry with directional flow resistors

**Fig. 2:**
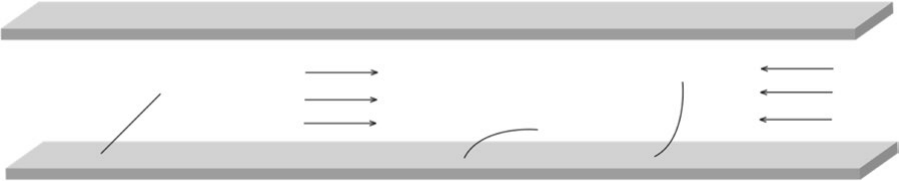
Orientation of cylinders to promote flow in the left to right direction. Left—cylinder orientation for zero flow. Middle—left to right flow flexes the cylinder so that it is oriented tangential to the flow. Right—right to left flow flexes the cylinder towards normal orientation. For simplicity, only one cylinder is shown, and it is attached to the wall, but in the solution in this section, cylinders are distributed throughout the channel. (From [17])

For the Sharp, et al. [17] solution, a periodic Darcy term is used to represent the drag imposed by cylinders that flex in response to flow direction (Fig. 2). To promote forward flow, the cylinders are tangential to forward flow, and normal to reverse flow. For simplicity, the cylinder in the figure is shown attached to the wall of the channel, but in the solution, cylinders are assumed to be distributed throughout the channel. (The model does not consider motion of the cylinders in the cross stream *y* direction.)

For this model, the mean flow rate is

6 $$\overline{Q }=1+\frac{-\Delta {P}_{\lambda }\left(\tau \right)-{\int }_{0}^{2\pi }\frac{{\gamma }^{2}\left(\xi \right)}{{H}^{2}\left(\xi \right)}\left[1-\frac{tanh\left(\gamma \left(\xi \right)\right)}{\gamma \left(\xi \right)}\right]d\xi }{{\int }_{0}^{2\pi }\frac{{\gamma }^{2}\left(\xi \right)}{{H}^{3}\left(\xi \right)}\left[1-\frac{tanh\left(\gamma \left(\xi \right)\right)}{\gamma \left(\xi \right)}\right]d\xi }$$

where $\Delta {P}_{\lambda }\left(\tau \right)\equiv \frac{2\pi {a}^{2}}{\lambda \mu c}\Delta {p}_{\lambda }$ is the normalized pressure difference over one wavelength λ, $\Delta {p}_{\lambda }$ is the pressure difference over one wavelength, *a* is the mean paraarterial gap height, μ is the dynamic viscosity, *c* is the wavespeed, $\gamma \equiv \frac{h\sqrt{K}}{2l}$ is the Darcy number, h is the periodic gap height, 2* l* is the spacing between cylinders, $H\equiv \frac{h}{a}=1+\phi cos\left(\xi -\tau \right)$ is the normalized periodic height of the paraarterial gap, $\phi \equiv b/a$ is the amplitude ratio of peristaltic wall motion, *b* is the dimensional amplitude of wall motion, $\xi \equiv 2\pi x/\lambda$ is the normalized axial coordinate and $\tau \equiv 2\pi ct/\lambda$ is normalized time. The drag coefficient K for cylinders is defined by a curve fit described by Sharp, et al. [17] and varies by a factor of 2 for normal versus tangential flow. The phase of the periodic drag function is shifted by *π*/2 compared to Sharp, et al. to promote antegrade rather than retrograde flow. While Sharp, et al. analyzed both sinusoidal and discontinuous switching of cylinder orientation, here switching is used since it promotes the greatest mean flow. The mean velocity across the cross section is given by Eq. 2.

## Results

**Fig. 3:**
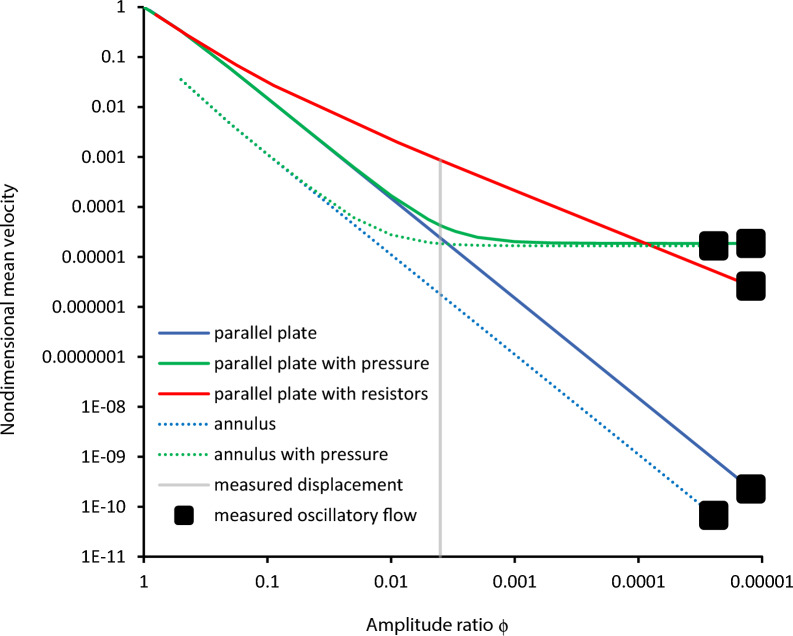
Mean flow rate normalized by the maximum peristaltic mean flow rate versus wall-amplitude-to-gap-height ratio

Figure 3 shows the mean velocity $\overline{U }=\overline{Q }$ for three different cases. The blue curve is for flow in the parallel-plate model with no imposed pressure gradient, $\Delta {P}_{\lambda }=0$. For the measured paraarterial gap amplitude ratio of ϕ = 0.004 (gray line on the figure), the mean flow rate is 0.000024, which is close to that measured (dimensional velocity of 24 μm/s versus measured 18.7 μm/s). However, the oscillatory velocity amplitude is too large at 4000 μm/s versus measured 12.3 μm/s. The Mestre, et al*.* [7] measurements quantified the displacement only of the inner wall of the channel. It stands to reason that the outer wall may also move, which would reduce the effective amplitude ratio. For an amplitude ratio ϕ = 0.00353, at which the measured mean velocity is matched, the velocity amplitude is still too large at 3530 μm/s. At amplitude ratio ϕ = 0.0000123, the measured peak velocity is matched, but the mean velocity is an insignificant 0.000227 μm/s (black square on the figure).

The effect of pressure difference in the parallel-plate channel is shown by the green curve in Fig. 3. As predicted by Eq. 1, pressure has little effect on the flow for large amplitude ratio, but becomes strong as ϕ decreases. The mean and oscillatory velocities are both matched with a nondimensional pressure difference of $\Delta {P}_{\lambda }=$ 0.000352 at an amplitude ratio of ϕ = 0.0000123 (the right-hand end of the green curve). This corresponds to a dimensional pressure difference of $\Delta p=\frac{\lambda \mu c}{2\pi {a}^{2}}\Delta {P}_{\lambda }\frac{L}{\lambda }=$ 1.56 mmHg, where the channel length is taken as *L* = 2 m.

The effect of flexing flow resistors approaches zero as ϕ approaches its maximum value, however, the effect increases more quickly with decreasing ϕ than that of a pressure gradient (Fig. 3). Mean velocity is matched at ϕ = 0.0000881, but the oscillatory velocity is too large at 88.1μm/s. At a smaller ϕ = 0.0000123 that matches the oscillatory velocity, the mean velocity is too low at 2.61 μm/s. The volume fraction of cylinders for these results is a somewhat arbitrary ${\varepsilon }_{0}=\frac{\pi {a}_{c}^{2}}{4{l}_{0}}=0.01$, where *a*_*c*_ is the cylinder radius and *l*_*0*_ is the mean cylinder spacing. The mean velocity is nearly insensitive to $\varepsilon$ at the amplitude ϕ= 0.0000123 that matches the oscillatory velocity, thus a simultaneous match of measured mean and oscillatory velocities is not possible.

The dotted blue curve in Fig. 3 presents the Wang & Olbricht [14] model for a nonporous (Φ = 1) annulus with zero pressure difference $\Delta {P}_{\lambda }=0$. The curve confirms that the annular model with oscillating inner wall has less potential for creating mean flow than the parallel-plate model. The gray line again shows the mean velocity for the measured displacement. For this model, the mean velocity is 2 μm/s with an oscillatory velocity amplitude of 2000 μm/s. For matched mean velocity at amplitude ϕ = 0.01223, the oscillatory velocity is too great at 6110 μm/s. For matched oscillatory velocity amplitude at ϕ= 0.0000246, the mean velocity is only 0.0000756 μm/s (black square).

With a pressure difference of 1.54 mmHg added (dotted green curve), both the mean and oscillatory velocities can be matched to measured values (right end of the dotted green curve), at an amplitude ratio of ϕ = 0000246.

**Fig. 4:**
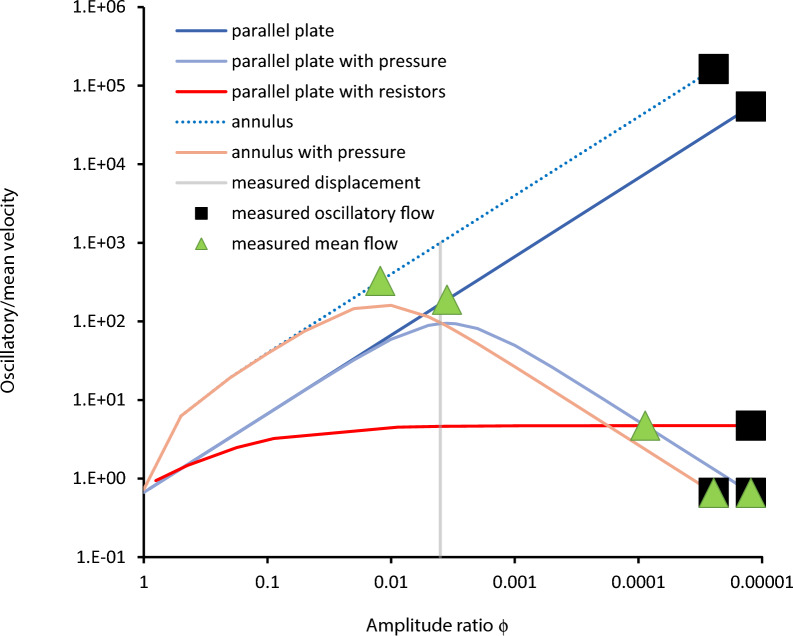
Ratio of oscillatory to mean velocities versus wall-amplitude-to-gap-height ratio for all models. This ratio is 0.66 for the Mestre, et al. [7] measurements

Additional evidence of the inherent inability of peristalsis alone to match the Mestre, et al*.* [7] data is presented in Fig. 4, which shows the ratio of oscillatory to mean velocity. For the Mestre, et al*.* [7] measurements, this ratio is 0.66. Figure 4 shows that at the measured displacement of ϕ = 0.004, the ratios are too large at 167 and 100 for the parallel-plate and annulus models, respectively, without a pressure gradient. Where the oscillatory velocity is matched to the measurements, the ratios are 54,200 and 163,000 for the parallel-plate and annulus models, respectively. Indeed, a ratio of 0.66 is approached for the parallel-plate model only as $\phi \to 1$, and for the annulus model the smallest ratio obtainable is 6.27 for ϕ = 0.5 (because the artery radius cannot collapse beyond its center). This fundamental characteristic of the performance of peristaltic channels reinforces that the mean flow must be driven by a different mechanism. With a pressure gradient in both the parallel-plate and annular models, the ratio initially rises for decreasing ϕ, but then decreases to 0.66 at smaller ϕ that simultaneously matches mean and oscillatory velocity. The parallel-plate model with directional resistors exhibits an asymptotic ratio for decreasing ϕ that is too high at about 4.7.

## Discussion

It bears emphasizing that no points on the curves of either model exist that can simultaneously match measured mean and oscillatory velocity in the subarachnoid paraarterial space without imposing a pressure gradient. Directional flow resistors improve the match, but the mean velocity remains about an order of magnitude too small when the oscillatory velocity is matched. Both models are long compared to the wavelength. Shorter channels, which may be more physiologic, would produce less mean flow. These results provide a clear indication that peristalsis alone cannot drive the paraarterial flow as measured by Mestre, et al*.* [7].

*Limitations of the solution*—The simplified geometries of the parallel-plate and annular models comprise an obvious and important limitation. The lengths of both models contain multiple wall displacement wavelengths, which promotes the effectiveness of the peristaltic motion in creating mean flow. (Romano, et al*.* [11], for instance, found that end effects extend a couple of wavelengths from both upstream and downstream ends, thus four or more wavelengths are necessary to approach fully-developed flow.) Even so, the predicted mean velocities where oscillatory velocity is matched to the measurements are four orders of magnitude too small. The more physiologic branching network can be expected to attenuate the arterial wall motion and add resistance at the branches, which would reduce mean flow even further. Downstream resistance of the parenchyma and the rest of the glymphatic circulation would also tend to decrease mean flow.

Compliance of the outer wall could explain the mismatch between the very large oscillatory velocity predicted with the measured arterial wall displacement versus the smaller measured oscillatory velocity. The scale of oscillatory velocity is dictated by wall displacement, thus it is clear that the effective amplitude ratio at the measurement site must be two orders of magnitude smaller than that calculated from arterial wall motion alone. As evident in Fig. 3, a smaller amplitude ratio by two orders of magnitude leads to smaller mean flow by four orders of magnitude.

It was argued by Ladrón-de-Guevara, et al. [8] that adding a Windkessel (parallel resistance and compliance) boundary condition promotes a match of mean and oscillatory velocity. Two orders of magnitude smaller oscillatory velocity is indeed predicted downstream of the compliance, with the bulk of the oscillatory flow going into and out of the compliance. For the measured mean velocity to apply in the MCA, there would need to be four or more wavelengths of channel upstream of the MCA to achieve fully-developed peristalsis. The internal carotid artery, which feeds the MCA after branching from the common carotid artery in the neck, is 9–11 mm long in mice and only part of it is in the skull [19]. The full length is only about one tenth of a wavelength. Therefore, it appears that peristalsis with small ϕ would not be effective. Large ϕ, closer to the positive displacement value ϕ= 1 might drive mean flow in spite of the short channel length. Note that the ratio of oscillatory to mean velocity approaches the Mestre, et al*.* [7] value of 2/3 as ϕ approaches 1 (Fig. 4). However, here the peak velocity approaches the wave speed. Peristalsis in the ICA with ϕ = 1 could match both velocities if the wavespeed were about four orders of magnitude slower and the wavelength about an order of magnitude shorter. Verification of paraarterial spaces surrounding the ICA, and measurement of fluid velocities and arterial and outer wall displacement are needed.

The details of outer wall motion remain to be measured. Substantial brain tissue deformation has been measured during neural vasodilation [18]. Recent measurements of outer and pial artery wall motion during low frequency (0.1–1 Hz)_oscillations during sleep in mice show that the two move more or less synchronously, with the outer wall having smaller amplitude [20]. This reduces the effective amplitude ratio, which tends to support the findings of this study. However, motion during higher frequency (~ 10 Hz) cardiac-mediated oscillations remains to be quantified.

The models presented here provide predictions of the required amplitudes, but phase is also important. A phase offset between arterial and outer wall waves was key to driving steady streaming in the Romano, et al*.* [15] model. Coloma, et al*.* [21] also found forward or reverse net flow, depending on the relative motion of the inner and outer walls of an annular space. Such phase differences were neglected in the models applied in this study.

The models used in this study assume that both walls are impervious. The effect of permeable walls has been studied by Kedarasetti, et al. [18] and Romano, et al*.* [15]. Flow through the outer wall downstream of the measurement location around the MCA [7] could enhance mean flow at the measurement site, so long as the outflow was not entirely returned later in the peristaltic cycle.

A small, steady pressure difference is sufficient in both models to match the mean velocity, while peristalsis drives primarily the oscillatory flow due to the low amplitude ratio. This result is consistent with previous studies [11–15, 22–24]. For example, Kedarasetti, et al. [13] found a 0.01 mmHg pressure difference over a 5 mm long idealized channel resulted in a velocity of 24.4 μm/s, which for fully developed flow at the Mestre, et al*.* [7] velocity in a 2 m long channel becomes 3.07 mmHg. Daversin-Catty, et al. [23], who simulated the paravascular space surrounding a realistic cerebral artery bifurcation, found a necessary pressure gradient of 1.5 mmHg/m, or 3 mmHg extrapolated to a 2 m channel. In this study, the required pressure differences are approximately half of these previous values, and are remarkably similar between the two models.

Even though it is small, the required pressure difference is much larger than the transmantle pressure difference [25, 26], available for inflow from and outflow to the subarachnoid space, which was the original hypothesis. More recent descriptions of the glymphatic system include possible outflow to cerebral lymphatics [3], which provides a greater overall pressure difference, at least for humans in supine posture (intracranial pressure 11.0 ± 2.1 mmHg versus lymphatic pressure of 0—1 mmHg) [27]. In upright seated posture, outflow to lymphatics does not help (71 degree tilt, seated posture, intracranial pressure − 1.8 ± 3.2 mmHg versus the same lymphatic pressure). Regardless, the source and mechanism of the necessary hydraulic pressure difference, as well as the anatomy of inflow and outflow, remains to be identified.

Injection of tracer fluid causes an increase in intracranial pressure (ICP) that is larger than that predicted by the models in this study to drive pararterial flow [28, 29]. A model suggests that the increase persists in mice for 17 min post-injection [5]. It is curious, then, that an improved injection/withdrawal protocol that does not increase ICP resulted in the same tracer velocities [29]. The Wang & Olbricht [14] model predicts that flows are increased in the radial direction from the site of injection, but not in the tangential direction. The MCA is more or less tangential to the cisterna magna. Injection into the cisterna magna in the brain is more complex than into a hydraulically isotropic material, but perhaps the direction of the paraarterial channels influences the lack of influence of pressure on the transport. Alternatively, the indifference to pressure may indicate that the observed mean transport is dispersive, not convective.

A more realistic elliptical model of the cross section of the paraarterial space reduces hydraulic resistance compared to an annulus [30], but also likely decreases the potential for peristaltic motion of the artery wall to drive mean flow. Therefore, a longitudinal pressure gradient would still be required, but it may be smaller.

An oscillatory pressure gradient can produce net flow in a peristaltic channel that is short compared to the wavelength [31], but this potential diminishes with increased channel length. The effects of osmotic pressure and facilitated water transport also remain to be investigated.

Directional flow resistors increase mean velocity, but in this model cannot create a match while also matching oscillatory velocity. The collapse of paraarterial spaces after death or during fixation [32] suggests that little structure exists within these channels. Nonetheless, a small volume fraction of such flexing structure might be difficult to detect. Given the variety and complexity of transmembrane proteins [33], it would not be surprising to find specialization according to hydraulic function, however, directional flow resistance has not yet been discovered.

## Conclusion

Peristalsis drives oscillatory flow that scales with the wall displacement amplitude ratio. Measured oscillatory velocity in the paravascular space surrounding the MCA is inconsistent with (too small for) the measured displacement of the arterial wall alone. Displacement also of the outer wall could provide a smaller effective wall displacement amplitude rato that creates oscillatory flow that matches that measured. In vivo measurements of outer wall motion could add valuable insight into the mechanisms of paravascular flow.

At realistic amplitude ratio, *i.e.,* that matching the oscillatory velocity, the cartesian Stokes and annular Darcy models both predict mean velocity much smaller than that measured. No states on the curves of peristaltic performance can simultaneously match the measured oscillatory and mean flows, regardless of amplitude ratio. These results indicate that a mechanism other than peristalsis is required to drive the mean flow. Directional flow resistors increase mean velocity where oscillatory velocity is matched, but is still about an order of magnitude too low. A longitudinal pressure gradient, on the other hand, is shown to be fully capable of providing the measured mean velocity. However, other possibilities to explain the observed tranport exist that remain to be investigated.

## Data Availability

Data and materials are available from the author.

## Dimensional variables

*a*: Mean half height of parallel-plate channel, full height of annular channel
*A*: Vessel radius
*a*_*c*_: Cylinder radius
*b*: Wave amplitude
*c*: Wave speed
*h*: Height of channel
*K*: Cylinder drag coefficient
*L*: Length of channel
*p*: Pressure
*q*: Flow rate
*R*_*1*_*, R*_*2*_: Mean inner and outer radii of annular channel
*t*: Time
*u*: *x* Direction velocity in the laboratory frame
*x*: Streamwise direction
*y*: Radial direction
*κ*: Permeability
*λ*: Wave length
*μ*: Dynamic viscosity
*ν*: Kinematic viscosity

## Subscripts

*c*: Cylinder
*L*: Over the length of the channel
*λ*: Over one wavelength

## Dimensionless variables

$\xi \equiv 2\pi \frac{x}{\lambda }$: Normalized flow direction coordinate
$$\tau \equiv 2\pi \frac{ct}{\lambda }$$: Normalized time
$H\equiv \frac{h\left(x,t\right)}{a}$: Normalized half height of channel
$$P\equiv \frac{2\pi {a}^{2}}{\lambda \mu c}p$$: Dimensionless pressure
$Q\equiv \frac{1}{ac}q$: Dimensionless flow rate in the laboratory frame
$\overline{Q }$: Spatial mean dimensionless flow rate in the laboratory frame
$U\equiv \frac{u}{c}$: Dimensionless *x* direction velocity

## Dimensionless parameters

$$R\equiv \frac{ac\alpha }{\nu }$$: Reynolds number
$\alpha \equiv \frac{2\pi a}{\lambda }$: Wave number (related to slope and curvature of channel)
$$\gamma \equiv \frac{h\sqrt{K}}{2l}$$: Darcy number
${\varepsilon }_{0}\equiv \frac{\pi {a}_{c}^{2}}{4{l}_{0}}$: Volume fraction of cylinders
$$\phi \equiv \frac{b}{a}$$: Amplitude ratio
$$\Phi$$: Porosity
